# Current Approaches to the Treatment of Hepatorenal Syndrome

**DOI:** 10.7759/cureus.119475

**Published:** 2026-11-11

**Authors:** César A Rosales

**Affiliations:** 1 Medicine, Hospital Nacional de Escuintla, Guatemala, GTM

**Keywords:** acute kidney injury, albumin, hepatorenal syndrome, liver transplantation, norepinephrine, terlipressin, vasoconstrictor therapy

## Abstract

Hepatorenal syndrome (HRS) is a life-threatening complication of advanced cirrhosis that requires early recognition, treatment of precipitating factors, and coordinated transplant assessment. This narrative review examines pharmacological treatment, fluid management, kidney replacement therapy (KRT), transjugular intrahepatic portosystemic shunt (TIPS), and liver transplantation in adults, with emphasis on HRS with acute kidney injury (AKI). Contemporary diagnostic criteria recognize that hepatorenal physiology can coexist with tubular injury or chronic kidney disease (CKD) and discourage a routine 48-hour albumin challenge. Clinical assessment should integrate perfusion, oxygenation, and congestion, with ultrasound used as an adjunct when available. Terlipressin with individualized albumin improves renal reversal in suitable patients, but a consistent overall survival benefit has not been established, and respiratory failure remains a major safety concern. Norepinephrine is an important alternative where appropriate monitoring is available; midodrine with octreotide has a limited fallback role. Albumin dosing should reflect serial volume assessment rather than a serum albumin target. Kidney replacement therapy supports patients with metabolic or volume complications, while evidence for routine shunt placement in acute HRS remains insufficient. Liver transplantation addresses the underlying disease, although kidney recovery is variable. Biomarkers may assist clinical phenotyping, but their use to guide treatment requires prospective validation. The review emphasizes timely diagnosis, individualized treatment, and distinguishing renal recovery from survival.

## Introduction and background

Hepatorenal syndrome (HRS) is a severe complication of advanced cirrhosis and portal hypertension. Splanchnic vasodilation, reduced effective arterial blood volume, and compensatory renal vasoconstriction contribute to declining kidney function. Although traditionally described as functional renal failure, the acute kidney injury (AKI) form of HRS (HRS-AKI) can coexist with tubular injury, proteinuria, or pre-existing chronic kidney disease (CKD) [1,2].

The historical terms 'type 1 HRS' and 'type 2 HRS' distinguished rapidly progressive from more indolent kidney dysfunction. Current terminology uses HRS-AKI, HRS with acute kidney disease (HRS-AKD), and HRS with chronic kidney disease (HRS-CKD), according to the timing and duration of dysfunction. Hepatorenal syndrome-AKI uses dynamic creatinine and urine-output criteria rather than the older requirement for progression to a high fixed creatinine threshold; HRS-AKI may also occur on pre-existing CKD [2,3].

Management combines treatment of precipitating factors, vasoconstrictor therapy with individualized albumin administration, and assessment for liver transplantation. The American Association for the Study of Liver Diseases (AASLD) practice guidance and European Association for the Study of the Liver (EASL) guidelines provide the clinical foundation, while subsequent evidence has refined diagnosis and drug selection [4,5]. Terlipressin improved verified HRS reversal in the CONFIRM (A Multi-Center, Randomized, Placebo Controlled, Double-Blind Study to Confirm Efficacy and Safety of Terlipressin in Subjects With Hepatorenal Syndrome Type 1)trial, but respiratory adverse events and the absence of an established overall survival advantage limit a simple efficacy-based treatment hierarchy [6].

The clinical question is therefore broader than whether serum creatinine (SCr) improves: which treatment offers a reasonable prospect of renal recovery without compromising respiratory status, cardiovascular stability, or transplant candidacy? The answer depends on the cause of AKI, the severity of liver disease, fluid balance, treatment availability, and the patient's goals. Guidance on vasoactive drugs and albumin reinforces the need to match treatment to the indication rather than using these agents indiscriminately in cirrhosis [7].

This narrative review examines diagnostic developments, comparative treatment evidence, safety considerations, kidney support, and transplant planning in HRS. It focuses on the distinction between renal recovery and survival and on clinical decisions in settings with limited ICU or transplant access [1,3,8].

## Review

Scope and approach to the literature

This narrative review draws on a structured literature search of PubMed/MEDLINE, Scopus, Embase, Web of Science, and the Cochrane Library, focused on recent English-language publications from January 2020 through June 2026. Earlier pivotal clinical trials and practice guidance were retained selectively for their historical or continuing clinical relevance. Search concepts combined "hepatorenal syndrome with terlipressin," "albumin," "norepinephrine," "midodrine," "octreotide," "kidney replacement therapy," "liver transplantation," or "transjugular intrahepatic portosystemic shunt."

Eligibility and Source Selection

Eligible sources addressed the clinical management of adults with cirrhosis and HRS, with emphasis on HRS-AKI. Original clinical studies, systematic reviews, meta-analyses, and clinical practice guidance were considered when they informed treatment selection, renal recovery, safety, survival, or transplantation. Publications unrelated to this population or clinical scope, those without relevant information, and duplicate or redundant reports were excluded. Pediatric and non-human studies were outside the clinical population of interest. Relevant narrative reviews, prescribing information, older guidance, and pivotal trials provided clinical and historical context. The synthesis considers clinical studies, evidence syntheses, and consensus recommendations on HRS diagnosis and management [2,3]. Terlipressin dosing and safety considerations are based on the United States prescribing information [9].

The initial database search identified 147 records. After removal of 18 duplicates, 129 records underwent title and abstract screening; 94 were excluded because they did not address HRS treatment in the population of interest. Thirty-five full-text reports were assessed for eligibility. Eighteen were excluded because of the population (n = 5), intervention (n = 4), absence of relevant outcomes (n = 3), publication type (n = 4), or duplicate patient population (n = 2). Seventeen publications were included in the qualitative synthesis. Figure 1 presents this selection pathway using a flow diagram adapted from the Preferred Reporting Items for Systematic Reviews and Meta-Analyses (PRISMA) 2020 template [10].

**Figure 1:**
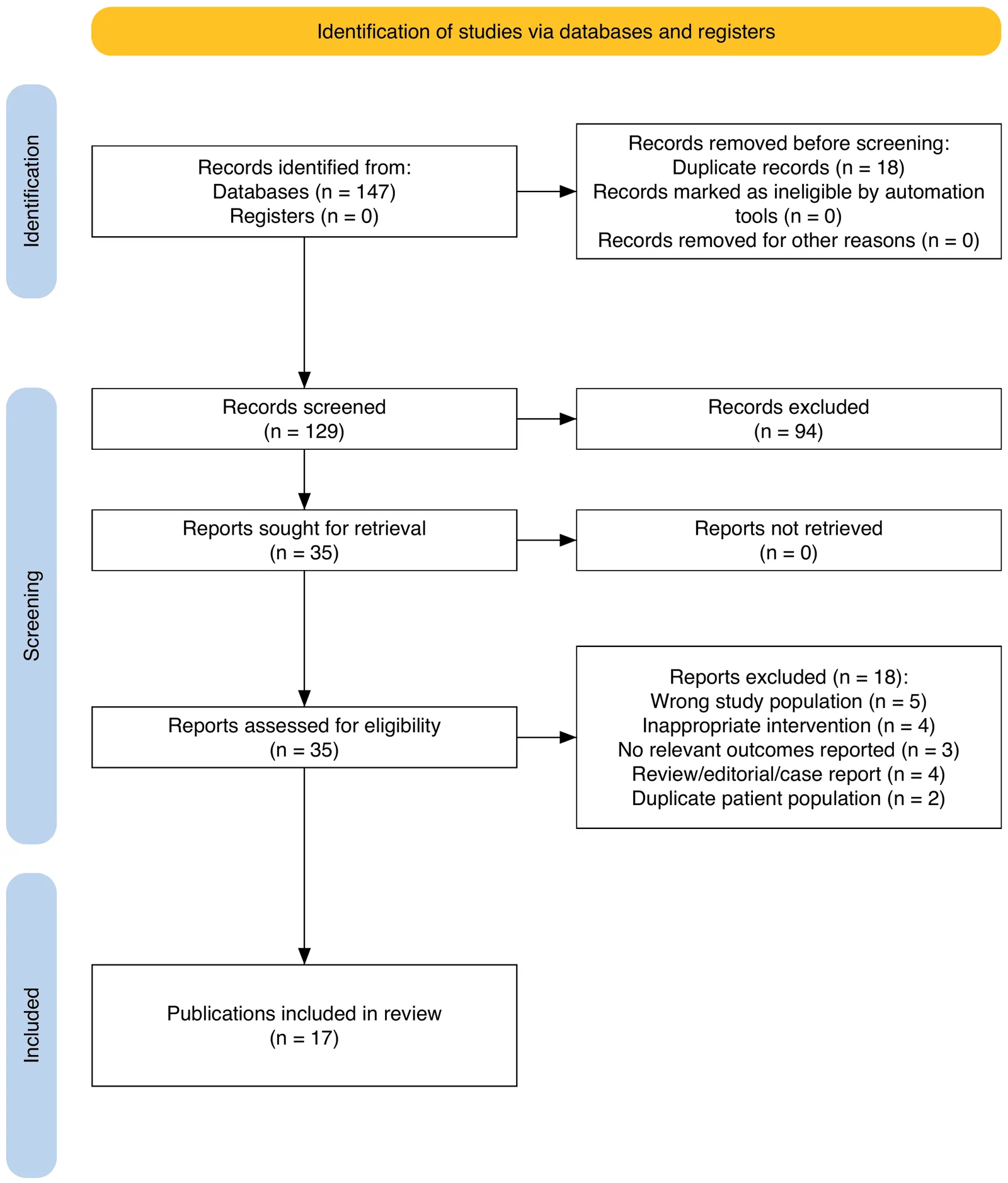
PRISMA flow diagram of the initial literature search Flow diagram adapted from the Preferred Reporting Items for Systematic Reviews and Meta-Analyses (PRISMA) 2020 template to illustrate publication selection for this narrative review [10].

Publications were screened for eligibility, and data were extracted on study design, population and HRS definition, intervention and comparator, albumin exposure, renal response criteria, follow-up, survival, and adverse events. Findings were synthesized qualitatively by treatment and clinical context. Effect estimates, confidence intervals, and p-values were drawn from the cited studies; no pooled estimates, meta-analysis, meta-regression, or formal grading of evidence certainty were produced.

Pathophysiology and diagnostic implications

Portal hypertension promotes splanchnic vasodilation and lowers effective circulating volume despite ascites and total-body fluid excess. Neurohormonal activation reduces kidney perfusion, while systemic inflammation and impaired cardiac reserve can aggravate the disturbance [1,8]. Renal venous congestion, cirrhotic cardiomyopathy, and intra-abdominal hypertension may also contribute to mixed AKI phenotypes. Thus, edema or ascites should not by itself determine whether additional volume is appropriate [3].

The 2024 Acute Disease Quality Initiative (ADQI) and International Club of Ascites (ICA) consensus identifies HRS-AKI in cirrhosis with ascites when SCr increases by at least 0.3 mg/dL within 48 hours or by at least 50% within seven days, and/or urine output is at most 0.5 mL/kg/hour for at least six hours. Diagnosis also requires no improvement within 24 hours after adequate volume resuscitation, when indicated, and no strong evidence that another process is the primary cause. The consensus discourages routine 48-hour albumin administration as a diagnostic prerequisite and uses HRS-AKI, HRS-AKD, and HRS-CKD according to duration [2].

Bleeding, infection, gastrointestinal fluid loss, nephrotoxic exposure, shock, obstruction, and intrinsic kidney injury require active evaluation. Urinalysis, urine sediment examination, appropriate imaging, and serial assessment of fluid status help identify overlapping mechanisms [2,4,5]. Underlying kidney disease does not rule out superimposed HRS-AKI, but clinicians should identify whether hepatorenal physiology is a predominant contributor rather than assign the diagnosis to every creatinine rise [2,3].

The practical aim is to avoid delayed treatment and unnecessary volume expansion. When resuscitation is clinically indicated, response should be reassessed within 24 hours; a routine 48-hour albumin challenge is inappropriate in patients who are euvolemic or congested. Bedside cardiac and lung ultrasound may complement examination, but isolated inferior vena cava measurements can be misleading in cirrhosis or intra-abdominal hypertension. No single ultrasound finding establishes HRS-AKI or fluid responsiveness [2,3].

Pharmacological treatment

Terlipressin

Terlipressin is a vasopressin analogue that counteracts splanchnic vasodilation. In the CONFIRM trial, 300 patients were randomized to terlipressin or placebo, with albumin strongly recommended in both groups. Verified HRS reversal occurred in 63/199 versus 17/101 patients (32% versus 17%; p = 0.006). This endpoint required two SCr measurements of 1.5 mg/dL or less, at least two hours apart, and survival without kidney replacement therapy (KRT) for at least 10 days after treatment. Overall survival was not improved; deaths attributed to respiratory disorders by day 90 occurred in 11% versus 2% [6].

The United States prescribing information specifies 0.85 mg of terlipressin by slow intravenous bolus over two minutes every six hours on days one to three; this is equivalent to 1 mg of terlipressin acetate. On day four, continue 0.85 mg every six hours if SCr decreases by at least 30% from baseline. If creatinine has decreased but by less than 30%, the dose may be increased to 1.7 mg every six hours. Discontinue treatment if creatinine is at or above baseline. Continue until 24 hours after two consecutive creatinine values of 1.5 mg/dL or less, measured at least two hours apart, or for a maximum of 14 days, unless an earlier safety-related discontinuation is required [9].

A randomized study of 78 patients found fewer adverse events with continuous terlipressin infusion than with boluses (35.29% versus 62.16%; p < 0.025), without a significant difference in overall response [11]. These findings support consideration of infusion protocols but do not establish universal superiority or interchangeability with the approved United States bolus schedule. Formulation, local approval, and institutional expertise remain relevant [3,9].

Respiratory assessment is central to patient selection. The United States label contraindicates treatment in hypoxia or worsening respiratory symptoms and in ongoing coronary, peripheral, or mesenteric ischemia. Do not initiate treatment in a hypoxic patient, including an oxygen saturation below 90%, until oxygenation improves. Use continuous pulse oximetry and discontinue terlipressin if oxygen saturation falls below 90% or respiratory symptoms worsen. The label advises avoiding use in grade 3 acute-on-chronic liver failure (ACLF) and states that patients with SCr above 5 mg/dL are unlikely to benefit. Assess volume status before treatment and adjust albumin and other fluids when congestion develops [9].

Treatment-related respiratory or ischemic complications can compromise transplant candidacy even when creatinine improves. Renal response, oxygen requirement, perfusion, and fluid balance should therefore be reviewed together, with interruption or discontinuation when harm outweighs the anticipated renal benefit [3,7,9].

Norepinephrine

Norepinephrine increases vascular tone and mean arterial pressure (MAP) and is an alternative when terlipressin is unavailable or unsuitable. The Acute Disease Quality Initiative and International Club of Ascites (ADQI-ICA) protocol uses continuous infusion at 0.5 to 3 mg/hour with ICU monitoring and central venous access, titrated to hemodynamic response and accompanied by individualized albumin [2]. Norepinephrine has a practical role in critically ill patients; use outside an ICU requires specialized staffing and monitoring [3].

In a meta-analysis of seven randomized trials involving 376 participants, Olson and Subramanian found inconclusive differences favoring terlipressin for HRS reversal (OR 1.33; 95% CI 0.80-2.22; p = 0.22) and short-term survival (OR 1.50; 95% CI 0.64-3.53; p = 0.26). These findings support norepinephrine as a reasonable alternative but do not demonstrate equivalence. Small, generally unblinded trials and differing diagnostic criteria limit interpretation; the review also reported industry funding and relevant consulting relationships [12].

Arora et al. randomized 120 patients with acute-on-chronic liver failure and HRS-AKI to terlipressin or norepinephrine. The HRS reversal favored terlipressin (40% versus 16.7%; p = 0.004), as did 28-day survival (48.3% versus 20%; p = 0.001). Drug-related adverse events occurred in 23.3% versus 8.3% (p = 0.02), whereas adverse events requiring discontinuation occurred in 15% versus 8.3% (p = 0.39). These distinct safety endpoints should not be conflated. The selected population and open-label design require caution when applying these results to other HRS-AKI settings [13].

Treatment selection also depends on monitoring capacity, vascular access, staffing, and availability. The consensus uses a MAP increase of approximately 10 mmHg above baseline to guide norepinephrine titration. Observational evidence supports consideration of larger increases in selected patients, although a universal target has not been validated. Hemodynamic response must be interpreted alongside kidney recovery, rhythm, and peripheral perfusion [2,3].

Midodrine and Octreotide

Midodrine with octreotide and albumin may be considered when preferred intravenous vasoconstrictors cannot be used. Easier administration outside an ICU does not establish an outpatient indication; patients still require close monitoring of kidney function, perfusion, precipitating illness, and fluid balance [4,8]. Cavallin et al. reported renal recovery in 19/27 patients receiving terlipressin versus 6/21 evaluable patients receiving midodrine and octreotide (70.4% versus 28.6%; p = 0.01) [14]. El-Desoki Mahmoud et al. likewise found greater full response with norepinephrine (15/26 versus 5/25; 57.6% versus 20%; p = 0.006), without a significant 30-day survival difference (42.3% versus 24%; p = 0.166) [15]. These findings support a restricted fallback role. A poorly effective midodrine-octreotide regimen may delay more effective treatment. When intravenous options are available and appropriate, treatment convenience should not determine selection [2,3].

Albumin

Albumin accompanies vasoconstrictor therapy for HRS-AKI, with the amount adjusted to volume status and cardiopulmonary tolerance. Its physiological and proposed non-oncotic effects should not be interpreted as proof of a survival benefit. During treatment, concentrated albumin is commonly given at 20 to 40 g/day with daily reassessment; it should be withheld when fluid overload or pulmonary edema develops [2,7].

The ATTIRE (Albumin to Prevent Infection in Chronic Liver Failure) trial randomized 777 hospitalized patients with decompensated cirrhosis to albumin targeting a serum concentration of at least 30 g/L or standard care. The protocol-defined composite of infection, kidney dysfunction, or death occurred in 29.7% versus 30.2% (adjusted OR 0.98; 95% CI 0.71-1.33; p = 0.87), with more severe or life-threatening adverse events in the albumin group. This was not a trial of established HRS-AKI treatment: it informs the risks of indiscriminate replacement rather than disproving the role of albumin with vasoconstrictors [16].

Fluid management requires distinguishing true volume depletion from congestion. Balanced crystalloids may be appropriate for volume loss, while albumin retains indication-specific roles in HRS-AKI, spontaneous bacterial peritonitis, and large-volume paracentesis. Once overload is recognized, fluid withdrawal and selected decongestive measures require individualized reassessment. The use of diuretics after hemodynamic stabilization is supported by limited observational evidence and should not be generalized as routine HRS-AKI treatment [2,3].

Interpreting the comparative evidence

Table 1 summarizes selected randomized trials of vasoconstrictors and albumin. Different HRS definitions, response thresholds, and follow-up periods preclude ranking treatments by cross-trial response percentages. 

**Table 1:** Selected randomized trials informing treatment decisions Albumin accompanied both vasoconstrictor arms in the comparative trials; in CONFIRM, it was strongly recommended in both groups. Results and p-values are reproduced from the cited original studies. Adverse events and events requiring discontinuation are separate outcomes. The ATTIRE trial provides indirect safety context rather than evidence of efficacy in established HRS-AKI. HRS: Hepatorenal syndrome; AKI: Acute kidney injury; ACLF: Acute-on-chronic liver failure

Study	Population and comparison	Main finding	Interpretive consideration
CONFIRM 2021 [6]	300 patients with historical HRS type 1; terlipressin versus placebo	Verified HRS reversal: 63/199 versus 17/101 (32% versus 17%); p = 0.006.	Renal endpoint improved; no established overall survival benefit
Cavallin et al. 2015 [14]	49 randomized patients; terlipressin versus midodrine and octreotide	Renal recovery: 19/27 versus 6/21 evaluable patients (70.4% versus 28.6%); p = 0.01.	Small trial; historical diagnostic criteria
El-Desoki Mahmoud et al. 2021 [15]	60 randomized patients; norepinephrine versus midodrine and octreotide	Full response: 15/26 versus 5/25 (57.6% versus 20%); p = 0.006. Survival difference not significant (p = 0.166).	Single center; small analyzed population
Arora et al. 2020 [13]	120 patients with acute-on-chronic liver failure and HRS-AKI; terlipressin versus norepinephrine	HRS reversal 40% versus 16.7% (p = 0.004); 28-day survival 48.3% versus 20% (p = 0.001).	Selected ACLF population. Drug-related adverse events: 23.3% versus 8.3% (p = 0.02); discontinuation: 15% versus 8.3% (p = 0.39).
ATTIRE 2021 [16]	777 hospitalized patients with cirrhosis; targeted albumin versus standard care	Composite outcome: 29.7% versus 30.2%; adjusted OR 0.98. 95% CI 0.71–1.33; p = 0.87.	General hospitalized cirrhosis population, not established HRS-AKI treatment; more severe or life-threatening adverse events with albumin.

Renal recovery can facilitate stabilization without ensuring survival, because liver failure, infection, cardiopulmonary complications, and transplant access influence prognosis. Wan et al. found greater treatment response with terlipressin than with placebo or albumin alone but no consistent survival advantage [17]. Comparisons of vasoconstrictors require consideration of the study population, response definition, volume exposure, and respiratory risk [3].

Table 2 summarizes treatment roles and separates interventions supported directly in HRS-AKI from procedures studied mainly for other portal-hypertension complications. Practical considerations include monitoring requirements, congestion, and ICU access [2,3].

**Table 2:** Clinical roles and limitations of treatment options Clinical roles are interpreted in relation to renal response, cardiopulmonary safety, and available monitoring [2,3]. HRS-AKI: Hepatorenal syndrome with acute kidney injury; TIPS: Transjugular intrahepatic portosystemic shunt; KRT: Kidney replacement therapy

Treatment	Clinical role	Principal consideration
Terlipressin with albumin	Preferred vasoconstrictor for suitable HRS-AKI patients [2,7].	Respiratory failure and ischemia; assess congestion and oxygenation; use formulation-specific dosing [3,9].
Norepinephrine with albumin	Alternative when terlipressin is unavailable or unsuitable; particularly relevant in ICU practice [2,3].	Close monitoring, staffing, vascular access, arrhythmias, and ischemia [2,3,12].
Midodrine and octreotide with albumin	Fallback when preferred agents cannot be used [4]	Lower renal response; avoid delaying an appropriate intravenous vasoconstrictor [3,14,15].
Albumin alone	Volume expansion for selected indications [7]	Not sufficient therapy for established HRS-AKI; serial volume assessment is required [2,3,7].
Transjugular intrahepatic portosystemic shunt (TIPS)	Selected portal-hypertension indications [5,18]	Recurrent-ascites evidence is contextual; not sufficient to support routine HRS-AKI treatment [2,5,18].
Kidney replacement therapy (KRT)	Metabolic and volume support; bridge or selected time-limited trial [8,19]	Clinical indications can arise at any stage; does not treat liver disease [2,3,19].
Liver transplantation	Definitive option for suitable patients [4]	Access and eligibility; kidney recovery is variable

Table 3 distinguishes United States labeling, consensus schedules, and trial protocols. Maximum duration is a stopping limit, not a reason to continue ineffective or poorly tolerated therapy. Doses and titration should follow the specified formulation and source protocol [2,3,9].

**Table 3:** Comparative doses and treatment duration for pharmacological regimens In the United States label, 0.85 mg terlipressin equals 1 mg terlipressin acetate [9]. The consensus recommends stopping for renal recovery, serious adverse reactions, lack of response despite maximal tolerated dosing, initiation of KRT, transplantation, or 14 days of treatment [2]. Its summary table gives 48 to 72 hours without improvement at maximal dosing; the accompanying consensus statement uses 48 hours. Formulation conventions and the chosen source protocol must be specified. Weight-based norepinephrine regimens and larger MAP increases have also been described in selected patients; these do not replace the consensus mg/hour schedule [3]. SCr: Serum creatinine; MAP: Mean arterial pressure; KRT: Kidney replacement therapy; HRS-AKI: Hepatorenal syndrome with acute kidney injury; ADQI-ICA: Acute Disease Quality Initiative and International Club of Ascites

Treatment and source	Route and dose	Dose adjustment	Duration or stopping limit
Terlipressin United States label [9]	IV bolus over two minutes: 0.85 mg every six hours on days one to three	Day four: continue 0.85 mg every six hours for a ≥30% SCr decrease. For a decrease <30%, the dose may be increased to 1.7 mg every six hours. Stop if SCr is at or above baseline.	Maximum 14 days; stop 24 hours after two consecutive SCr values ≤1.5 mg/dL, at least two hours apart.
Terlipressin ADQI-ICA consensus [2]	Continuous IV infusion: 2-12 mg/day, as reported in the consensus	Increase by at least 2 mg/day every 24 hours if SCr has not fallen by 25%; maximum 12 mg/day.	Maximum 14 days; discontinue earlier according to response or stopping criteria.
Norepinephrine ADQI-ICA consensus [2]	Continuous IV infusion: 0.5-3 mg/hour, with albumin	Reassess every four hours. Increase by 0.5 mg/hour as tolerated if MAP has not risen by approximately 10 mmHg or urine output remains inadequate, to a maximum of 3 mg/hour. Monitor perfusion and rhythm.	Maximum 14 days under the consensus stopping framework; stop earlier when indicated.
Midodrine plus octreotide ADQI-ICA consensus [2]	Midodrine: oral 7.5-15 mg every eight hours. Octreotide: subcutaneous 100-200 μg every eight hours. Add albumin.	Individualize within the reported dose ranges according to hemodynamic response and tolerance.	Maximum 14 days under the consensus stopping framework; stop earlier when indicated.
Midodrine plus octreotide El-Desoki Mahmoud trial [15]	Midodrine: oral 5 mg three times daily, up to 12.5 mg three times daily. Octreotide: subcutaneous 100 μg every six hours, up to 200 μg every six hours.	The trial used the indicated escalation ranges, with IV albumin 20-40 g/day.	Maximum 10 treatment days. The 30-day survival assessment was follow-up, not treatment duration.
Albumin during HRS-AKI treatment, ADQI-ICA consensus [2]	IV albumin (20-25%): Usually 20-40 g/day with a vasoconstrictor	Reassess daily; withhold for volume overload or pulmonary edema.	During vasoconstrictor therapy as tolerated. A separate fixed course is not specified.
Albumin historical cavallin trial regimen [14]	IV 1 g/kg on day one, then 20-40 g/day	Trial protocol: an initial loading dose is not mandatory for every contemporary patient.	Loading on day one, then daily adjunctive dosing during treatment; assess current fluid status before applying this regimen.

Non-pharmacological treatment

Transjugular Intrahepatic Portosystemic Shunt

Transjugular intrahepatic portosystemic shunt (TIPS) decompresses portal hypertension, but evidence from ascites should not automatically be extrapolated to acute HRS. Bureau et al. randomized 62 patients with cirrhosis and recurrent ascites to covered TIPS or repeated large-volume paracentesis with albumin; one-year transplant-free survival favored TIPS. The trial addressed recurrent ascites and does not establish efficacy for rescue treatment of contemporary HRS-AKI [18]. It may be considered for an established portal-hypertension indication after multidisciplinary assessment of liver reserve, encephalopathy, cardiac function, pulmonary vascular disease, and infection. The evidence does not support routine TIPS placement for HRS-AKI [2,5].

Kidney Replacement Therapy

Kidney replacement therapy provides support for refractory hyperkalemia, acidosis, pulmonary edema, or uremic complications. It does not correct the underlying liver disease, and initiation should be based on the clinical context rather than creatinine alone. It can be required at any stage of HRS-AKI care; hemodynamic instability may favor a continuous modality, although a survival advantage over intermittent dialysis has not been established [2,3,8].

Transplant ineligibility does not make KRT universally inappropriate. McLean Diaz et al. studied 372 transplant-ineligible patients with cirrhosis and AKI and reported high mortality and substantial use of intensive end-of-life care. The cohort included HRS-AKI and acute tubular necrosis, so its findings are not exclusively HRS-specific. Its observational design cannot establish a causal dialysis benefit. Decisions should consider reversibility, organ failures, patient preferences, and a time-limited trial with defined reassessment goals when appropriate [19].

Liver Transplantation

Liver transplantation addresses the underlying hepatic disease and remains the definitive therapeutic option for suitable patients. Referral should occur alongside HRS treatment rather than after all pharmacological options have failed. A creatinine response does not eliminate the need for transplant assessment, because the patient's liver disease and risk of further decompensation persist [4,5].

Kidney recovery after liver transplantation is variable. Prolonged kidney dysfunction, structural injury, and pre-existing kidney disease may alter the likelihood of recovery and the choice between liver transplantation alone and simultaneous liver-kidney transplantation. This decision requires specialist assessment and the allocation rules applicable to the transplant program. Evaluation should also address infection, nutrition, frailty, cardiopulmonary fitness, and other modifiable risks that affect candidacy [20].

Integrated Clinical Management

Clinical care should identify the predominant AKI mechanism, treat reversible precipitants, initiate an appropriate vasoconstrictor-albumin plan promptly, and assess transplantation in parallel. Repeated evaluation of perfusion, respiratory status, and congestion should guide fluid administration [3]. Figure 2 presents this sequence and the concurrent assessment of KRT needs and transplant candidacy.

**Figure 2:**
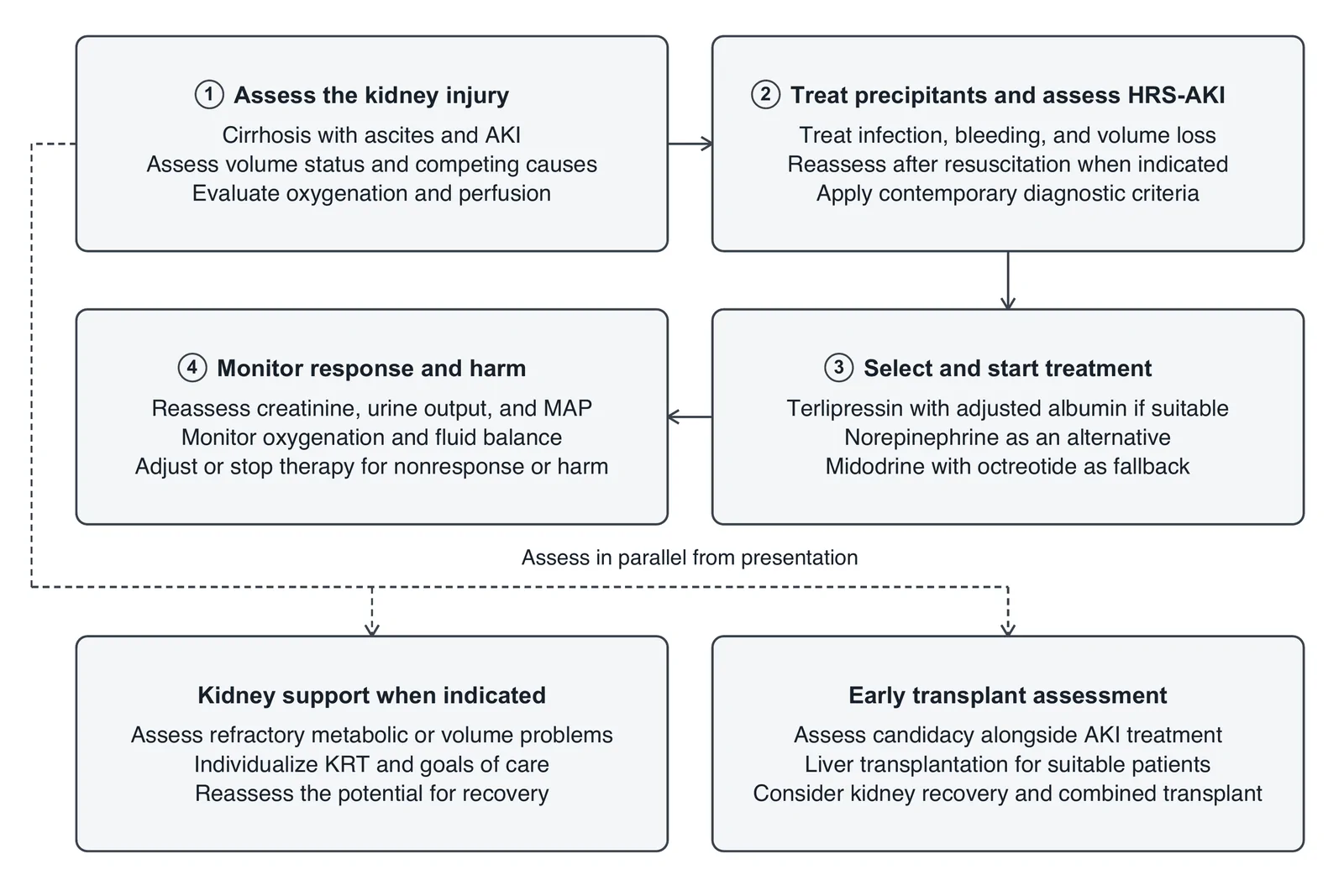
Clinical management of HRS Clinical schematic synthesizing the approach discussed in this review [2-4,7-9]. Solid arrows indicate the main assessment and treatment sequence; dashed branches indicate concurrent assessment of kidney support and transplant candidacy from presentation. Kidney replacement therapy is initiated for clinical indications, not automatically at presentation.  The schematic is not a validated decision rule. HRS: Hepatorenal syndrome; AKI: Acute kidney injury; MAP: Mean arterial pressure; KRT: Kidney replacement therapy

Nonresponse should prompt reconsideration of diagnosis, dose adequacy, fluid tolerance, and the objective of continued treatment. Escalation, transfer, and kidney support should be discussed with hepatology, nephrology, and critical-care teams when available [2,3,19].

Biomarkers and emerging therapies

Serum creatinine can underestimate kidney impairment in cirrhosis because of sarcopenia and altered creatinine generation. Cystatin C and urinary injury biomarkers may complement assessment. Gambino et al. support a diagnostic and prognostic role for urinary neutrophil gelatinase-associated lipocalin, but overlapping values and mixed AKI mechanisms limit a binary separation of HRS from structural injury [21]. Fractional sodium excretion and other isolated markers also require interpretation within the clinical context [3].

The 2026 systematic review by Chen et al. reinforces interest in biomarkers while highlighting the range of diagnostic approaches. A marker associated with injury or mortality is not automatically a validated treatment-selection tool. Assay standardization, thresholds, external validation, and availability remain important considerations. Prospective studies must show that adding a biomarker changes management and improves outcomes before biomarker-guided therapy can be regarded as standard care [22].

Extracorporeal liver support and other investigational treatments require evaluation against clinically meaningful endpoints rather than creatinine reduction alone [1]. Alternative vasopressors or vasopressor combinations discussed in critical-care practice have limited HRS-specific evidence and should not be presented as established substitutes for terlipressin or norepinephrine [3].

Limitations and future directions

This narrative review provides a qualitative synthesis of selected literature, and relevant evidence may have been missed. Risk of bias within individual studies was not formally assessed. No new pooled effect estimates or formal grading of evidence certainty were produced. Evolving HRS definitions, different response criteria, heterogeneous albumin exposure, and variable follow-up limit direct comparisons. Findings in HRS-AKI should be extrapolated cautiously to HRS-AKD or HRS-CKD.

Future research should prioritize comparative studies in well-characterized contemporary populations, individualized approaches to albumin administration that optimize safety, and improved identification of patients most likely to benefit from vasoconstrictor therapy. Trials should evaluate renal recovery alongside respiratory adverse events, KRT use, transplantation outcomes, and survival. Prospective evaluation of biomarker-guided management could inform decisions regarding KRT and combined liver-kidney transplantation. Including settings with limited access to intensive care and transplantation would broaden applicability.

## Conclusions

Hepatorenal syndrome management requires prompt recognition, treatment of precipitating factors, and early transplant assessment. Terlipressin with individualized albumin improves renal reversal in suitable patients; norepinephrine is an important alternative, while midodrine with octreotide has a limited fallback role. Perfusion, oxygenation, and fluid tolerance should guide therapy because renal recovery does not ensure survival. Kidney replacement therapy provides support when indicated, and TIPS requires selection for established portal-hypertension indications. Liver transplantation addresses the underlying disease. Management should be individualized according to the patient's kidney-injury phenotype and clinical course.
